# Violence against women and mental disorder: a qualitative study in Bangladesh

**DOI:** 10.1186/s41182-018-0085-x

**Published:** 2018-03-01

**Authors:** Md. Manirul Islam, Nasim Jahan, Md. Delwar Hossain

**Affiliations:** 1 Training Unit, icddr,b, 68, Shaheed Tajuddin Ahmed Sarani, Mohakhali, Dhaka, 1212 Bangladesh; 2 Department of Psychiatry, BIRDEM General Hospital and IMC, Dhaka, Bangladesh; 3 National Institute of Mental Health, Sher-e-bangla Nagar, Dhaka, 1207 Bangladesh

**Keywords:** Violence against women, Mental disorders, Bangladesh, Qualitative study

## Abstract

**Background:**

Violence affects 15–75% of women across the globe and has a significant impact on their health, well-being, and rights. While quantitative research links it to poor mental health, there is a lack of qualitative enquiry in how women experience it, and how it is related to the mental disorders in Bangladesh. This information is important in understanding the situation and structuring a locally appropriate and culturally sensitive program.

**Methods:**

We adopted a phenomenological approach and conducted 16 in-depth interviews, three informal interviews, one focus group discussion, and one key informant interview. We also reviewed published reports and documents. We followed criterion sampling in selecting women with mental disorders who experienced violence. We explored their experiences and understanding of the issues and described the phenomenon.

**Results:**

We found that Bangladesh society was largely controlled by men, and marriage was often forced on women. Women often were blamed for any mishap in the family and married women were under social and emotional pressure to keep the marital relationship going even when painful. We found all forms of violence (physical, emotional, sexual etc.) and most of the time found more than one type in women with mental disorders. Sexual violence is a reality for some women but rarely discussed. We found the society very tolerant with mental disorder patients and those who resorted to violence against them.

We identified four theoretical understandings about the role of violence in mental disorders. Sometimes the violence predisposed the mental illness, sometimes it precipitated it, while other times it maintained and was a consequence of it. Sometimes the violence may be unrelated to the mental illness. The relationships were complex and depended on both the type of mental disorder and the nature and intensity of the violence. We found most of the time that more than one type of violence was involved and played more than one role, which varied across different types of mental disorders. Interestingly, not all violence that mentally disordered women faced was because they were women, but because of mental disorders, which brought violence to them as a consequence.

**Conclusions:**

The findings of this first ever qualitative study into the experiences of violence by women with mental disorder in Bangladesh can be used in developing a culturally specific intervention to reduce both violence and mental disorders in women.

## Background

Violence against women means “any act of gender-based violence that results in, or is likely to result in, physical, sexual or psychological harm or suffering to women, including threats of such acts, coercion or arbitrary deprivation of liberty, whether occurring in public or in private life” [1]. It affects 15–75% of women across the globe and is a significant threat to their health, well-being, and rights [2–4]. A multicenter study in 11 countries in the world has found that around 15–71% of women faced physical and sexual violence and 20–75% emotional abuse in their lifetime. In Bangladesh, between 50 and 70% of women face some form of violence, with the prevalence varying across different settings [5, 6].

Violence against women is associated with a number of mental health problems including mood, anxiety, post-traumatic stress, and somatoform disorders [7–10]. World Health Organization (WHO), in a mixed method research, reported violence as an important factor of poor mental health in women across countries including Bangladesh [5]. García-Moreno et al. in the multicenter study referred to earlier mostly focused on intimate partner violence and its impact on mental health, reported as emotional distress as a part of different dimension of health. They did not address violence committed by others nor did they specifically look into its relationship with mental disorders. A quantitative study in nine low- and middle-income countries found a link between violence with suicides [9]; up to 90% of people may have some form of mental disorder at the time of suicide.

Studies across India and Pakistan found an association between domestic or intimate partner violence, and poor mental health including suicidal ideation [11–13]. Like most others, the studies were quantitative and related the symptoms of poor mental health, rather than examining specific mental disorders.

In a quantitative study in Bangladesh, Naved and Akhtar found that suicidal ideation was more common among women facing physical or emotional violence compared to women who were not facing such violence [14]. However, they also did not specifically focus on different types of mental disorders as a consequence of violence against women nor did they explore the experience of women facing such violence and how it was associated with different types of mental disorders.

Mental disorders are multi-factorial. Several factors often interact in a complex way to cause and or maintain mental disorders; some related to gene and environments predispose an individual in early childhood while others may occur before the disease to precipitate it. Some factors may maintain it. A number of researchers underscored the importance of understanding how violence against women affects their mental health [2, 15]. The exploration is important because the nature and impact of violence against women vary across cultures. For example, two thirds of women physically abused in Bangladesh did not tell anybody about the violence while about 80% in Brazil and Namibia city did so [5]. The lack of sharing may result in a higher magnitude of mental disorders in women in Bangladesh. In a study carried out in an urban community, around half of adult women reported to have mental disorders [16].

Understanding the mental health problems in women is important because of their increasing participation in economy; their essential roles in growth and development of children and welfare of the elderly [7]. If we want to structure any culturally specific and “internationally instructive” interventions aimed at preventing mental health problem associated with violence among women, we need to explore the experience of women who face violence and understand how it is related to their mental disorder [7, 15].

Qualitative research is an appropriate approach in exploring the experience of women facing violence and its association with mental disorder because of the complex nature of mental disorders and the multifaceted role of various factors including violence in predisposing, precipitating, and maintaining it [17–21].

## Methods

We adopted a phenomenological approach to understand and describe the experience of women who faced violence and were suffering from mental disorders. We used a modified Bengali version of the Humiliation, Afraid, Rape, and Kick (HARK) questions to identify women who faced violence and were clinically diagnosed by a mental health professional at the National Institute of Mental Health (NIMH) to determine their mental disorders. We adopted criterion sampling to identify the respondents. We included the respondents with mental disorders who answered “yes” to at least one HARK question and agreed to participate in the study. We chose HARK questions because of its high sensitivity of 81% (95%C.I. 69 and 90%) and specificity of 95% (95%C.I. 91 to 98%) with the cut-off score of ≥ 1 [22]. We initially decided to conduct around 10 interviews to understand and describe the experience of women with mental disorders facing violence; with this number considered adequate for a phenomenological approach [23]. However, we eventually continued to 16 in-depth interviews (IDI) and three informal interviews to achieve maximum saturation. We collected data from patients often complemented by caregivers and from guardians only when the subject was not fully communicable. The median interview time was around 56 min; the shortest one was 39 min while the longest one was of 140 min. All interviews were carried out either by qualified or trained female and or male psychiatrists with postgraduate education. All formal interviews and discussion were carried out at different suitable places of NIMH. Table 1 shows the age, occupation, education, and psychiatric diagnosis of 16 patients involved in in-depth interviews and three patients involved in informal interviews.

**Table 1:** Demographic and type of mental disorders of the patients of interviews

Age in years (f^1^)	Occupation (f)	Years of schooling^2^ (f)	Diagnosis^3^ (f)
15–20 (8^4^)	House wife (8)	0–5 (1)	Depression and related (4)
21–25 (1)	Teacher (2)	6–10 (10)	Bipolar mood disorders (4)
26–30 (4)	Student (6)	11–15 (5)	Obsession (1)
31–35 (4)	Legal counsel (1)	16–20 (2)	Acute stress disorder (1)
36–40 (2)	Unemployed (2)		Conversion disorder (3)
			Schizophrenia (5)
			Mental retardation (3)
			Conduct disorders (1)

Notes: ^1^Frequency or how many; ^2^Information on years of schooling is missing for one respondent; ^3^Three respondents had dual diagnosis, ^4^Two participants were 15 years old and two 17 years old

We also conducted a focus group discussion (FGD) to get a wider view and triangulate our findings from the in-depth interviews. A total of five physicians, (two women and three men) who were involved in the treatment of patients with mental disorder attended the discussions which lasted a little more than 2 h. The corresponding author moderated the discussion. He also conducted one key informant interview (KI) with a leading psychiatrist in Bangladesh with around 20 years experience to triangulate and validate our earlier findings in IDIs, to capture missing information in the development of theoretical understanding. We used separate interview guides for KI and FGD. We used a judgmental sampling technique in selecting KI and participants for FGD. We also reviewed documents such as magazines, news reports, and other local research reports. We then compared them with our data to cross check and validate our understanding, which contributed to the credibility of our research [24]. The richness of the data (including from patients, psychiatrists, document reviews, and our own familiarity with the context and culture and experience in treating mental disorder patients) allowed us to construct and describe “truly” reflected “essence” of the phenomenon of violence against women and its relationship with mental disorders.

We recorded the interviews and discussion. We then listened to the recorded data to have a “sense of whole” [25] and identified units or segments relevant to our study aims. We took notes on our reflection while listening to the data as we understood it. We translated related verbatim quotes into English and wrote them along with the relevant units and notes with the time locator and file name underneath. That allowed us to track down the segment in the audio recording if needed. The segments were the “meaning units” that constituted the various aspects of “the essential structure” of the whole phenomenon [25]. We decided not to transcribe the data verbatim because of our previous experience where we found typing in local language difficult and contracting out the task of transcription to a Bengali typist not involved in research was ineffective. This was because of difference in accent, choice of words used, and educational background of the participants and transcriber’s failure to retrieve cue related information [26, 27]. We listened to the data and reread our notes and placed them all under broad categories. The segmentation and categorization of relevant data gave us “meaning units” of our reflection and quotes to describe “essence” of the phenomenon and allowed us to skip formal coding [25, 27]. We constantly compared categories and looked for the patterns and themes as it emerged during analysis. We summarized, revised, and structured our understanding of various thematic areas of the phenomenon through our insights against the context [27]. Finally, we describe the phenomenon as we understood it with text, diagram, and quotes.

### Ethical issues

We used a Bengali consent form and informed the respondents of their right to withdraw from the study at any time even after consenting. We specifically mentioned, “You can leave the discussion (interview) at any time. If you do not take part in the study it will not hamper your treatment” to allay “fear, subconscious repression, and concealment” [28]. We informed participants that they can withhold any information they do not want to disclose and assured them of confidentiality. We obtained verbal consent before interviewing an individual and recorded the consenting process for all IDIs, KI and FGD. We secured assent of the participants who were minor (aged less than 18 years) along with the consent of their mothers and in one case foster mother. We maintained confidentiality in every step of the study. We identified all recorded interview files with a unique code, understandable only to the corresponding author. We replaced the real name with a pseudonym in the article and secured data with the investigators. We were either qualified or trained in psychiatry that allowed us to offer necessary treatment and advice to those in need at NIMH. We obtained formal ethical clearance from the National Institute of Mental Health, Bangladesh.

## Results

It is important to have “rich contextualized descriptions” in a phenomenological approach [29]. We will first describe the context, then the phenomenon of violence against women with mental disorders and finally our understanding of the relationship between violence against women and mental disorders.

### The context

The society was male dominated and women often were blamed for any mishap in the family. Marriage in this society was a universal phenomenon. We found that parents were under great social pressure to marry off their daughter even when they were not grown up. Child (aged less than 18 years) marriage or giving dowry (giving cash or other assets by families of brides to grooms and or their families during the event of marriage) [30], although illegal, was practiced widely. We found it very common among the low socioeconomic groups. A family married off a girl when she was studying at sixth grade. Her mother narrated her experience of why they married off their daughter so early:

Their family was good, they are showing interest in the girl, do not want anything, and said, “we want the bride only”. Then [we] thought we are poor people, marrying her off will need money. Because they are saying [they do not want dowry] and have building [economically better of], let’s marry [her] off. The place, where we were tenant, the land lady also pursued. Then [we] married [her] off.

We found married women under social, and emotional obligation for keeping up the marital relationship even if it was painful; a divorcee was considered to be a “bad girl” and with “bad character”. A professional woman earnestly longing for her husband in spite of his brutal behavior, divorce and sue described her experiences: “That [I] was married to him; I am his wife, just this much; anything else is not needed. He, keeping me with, can marry five more. I’m his wife, I want only this identity.”. The pressure might mount up if the social status of the woman was high. She added: “My father was a village arbitrator, still he is. He used to mediate all the conflicts in the village. If such an event [divorce] happens in my father’s house, how will he show his face [in the society].”.

We found corporal punishment by mom and dad often an acceptable way of disciplining children or adolescent. Women often accepted violence as usual, in particular if it was by their husbands or parents. We found the phenomenon deeply rooted in the tradition. A discussant narrated his experience where he came across a father in law visibly disappointed with the village arbitration meeting for the violence committed by his son:

The father in law said, “I used to beat my wife but was not subjected to arbitration. Why is so much this arbitration for now?” [Interviewer: It means cultural factor is an issue]. The other thing is [the tradition of beating wives] has been continuing generation after generation, is it right? Father in law says, “I have beaten my wife, my son has beaten daughter in law, what’s wrong with this”.

In the absence of a social security system, parents in their old age often live with their sons or daughter. That often created a psychological conflict with in laws. The in laws often influenced the relationship between husband and wife and contributed to violence.

We found a transition in life style meant more women were venturing out in career oriented jobs and often spent more time with their colleagues than their husbands. Easily accessible mobile and social networking sites along with career oriented exposure often raised the possibility of pre or extramarital affair and related violence. A discussant narrated how a lady had a physical fight with her husband over her extramarital affair and attempted suicide and was taken to the hospital: “The lady, has children, has a husband, has everything but she is involved in extra marital affair. How has she done it? Through internet [face book].”. We found adolescents or youth often were sexually harassed using mobile or face book to develop a relationship. If fail, they did not oblige, men sometime circulate private photographs or distorted images as revenge. Consequently, parents and their daughters underwent tremendous psychological stress. Parents often stopped their daughters’ education and married them off early to avoid further unwanted situation. A psychiatrist narrated his experience:

Some girls, young girls who go to school, in the villages, in urban areas, while going to school, some *bokhate* [derailed] boys eve teases [sexually harassed] on the way to school, do eve teasing, make suggestive comments, force to stop going to school, [and] give proposal for marriage.

### The experience of violence and mental disorder

We found different types of violence such as using swear words, pushing, bashing, slapping, kicking, knocking, injuring head, hitting with a stick, throwing objects at, not giving food or family expense, living separately, taking away children, involving in extra marital affair or second marriage, humiliating arbitration, and violent sexual behavior rendered to the patients with mental disorders. Participants often reported more than one category of violence. A law professional, narrated her experience of violence she faced from her husband:

Most of the time, he hurled abusive words. [Interviewer: used abusive words, what else?]. Suddenly, he beats. Because of the beating my head struck a wall [showing how her head struck the wall], fell down. After falling down [he] poured water on my head [it is a culturally acceptable home remedy for the sick]. That is what. [he] used to hit my head most of the time.

We found sexual violence a reality but a taboo. No respondents voluntarily shared their experience of sexual violence they faced. An educated woman confessed previous sexual abuse at the last part of the interview when she was in a good rapport with the interviewer. She also shared how her two and half year old daughter was about to be sexually violated when she went to the wash room leaving her alone outside:

In front of my own eye, I was just coming down[from the wash room], saw that [he] is removing trouser, my kid’s trousers. [Interviewer: who?] That employee. This little kid, only two and a half years old. [Interviewer: Is it girl?] Girl. Has removed trouser and his, I was about to have a churn in head. I went [there] quickly. He left away, saying nothing, left with head down cast.

Sexual abuse most of the time was not reported because the perpetrators were often in close relationship with the victims. A little girl could be sexually abused by the nearest ones who were beyond any suspicion such as uncle in law, uncle, and brother in law, or house tutor. The victims and their families often wanted to keep it secret because exposing the incident would defame not only the victims but also erode family honor. This has profound implication on the development of mental disorders even in later life. Our key informant shared his experience:

Many girls who faced violence in early childhood, sexually abused, when grown up, many shared with me, “I have faced such an event in my life long time ago, after that I have problems, after that the event comes to my brain and mind”

We found the society very tolerant with mental disorder patients and also inactive against the ones who beat, yelled or threw stone or even burning matchstick at them. The following conversation with an intellectually disabled girl with conduct disorder, who was burnt with a matchstick thrown at her by someone, narrated the phenomenon:

Girl: Then my body was on fire, and my clothes.

Mother: Half of the shirt burnt

Interviewer: When caught on fire, did you not see [the perpetrator]?

Girl: No.

Mother: Thrown [stick] lit with match.

Girl: Thrown [stick lit] with match.

Interviewer: Thrown a matchstick? Were people around, what did they say? Did they say nothing?

Girl: They poured water on my body.

Interviewer: They poured water then, has he been caught? Has he been told anything?

Girl: No.

We also found the society self-centered and not aware about role of violence and its relationship with mental disorder. Poor access to grossly inadequate mental health services only adds to the suffering of mentally disordered women facing different types of violence. An unmarried woman with depression vividly described her situation:

I cannot forget, sir, cannot forget at all. I cannot forget anything, sir [crying];… my torture, that boy’s [she has relation with] torture, my family’s torture. I am sick; I cannot sleep on bed, cannot eat, [and] cannot sleep. Where will my life go? …I only want to live. I cannot take any more, my body cannot cope anymore.

### The relationship between violence and mental disorders

**Fig. 1:**
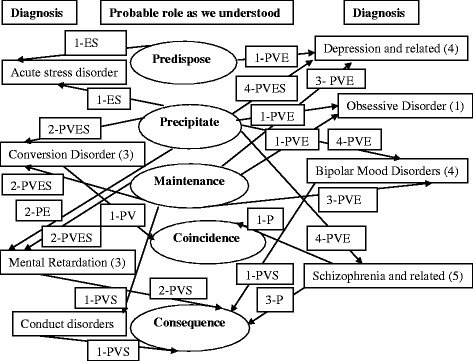
Role of different types of violence in different types of mental disorders as we understood. ***P*** physical, ***S*** social, ***V*** verbal, ***E*** emotional or psychological. Note, the numbers indicate the count of patients for descriptive purpose, the arrows indicate towards the types of relationship while the types of violence were placed on the arrows to illustrate what types of violence were associated with that relationship

We identified several patterns of relationship between violence against women and mental disorders. We found violence as a predisposing factor in some while in many a precipitator or maintenance factor. Sometimes, it was a mere coexistence and not perceived as unusually troublesome by the women with mental disorders. However, in many, it came as a consequence and often contributed to its maintenance. The type of violence and its severity perceived by women facing it sometimes played an important role in the development of a particular mental disorder. In the absence of adequate mental health facilities, women with severe and chronic mental disorders almost always faced some form of violence. It occurred when force was applied to restrain them from causing harm either towards self or others, it also happened when long exhausting caregiving took a huge toll on the care givers. Even the near ones themselves were either involved in perpetrating violence or kept silent when others were doing it. As shown in the Fig. 1, we found most of the time more than one category of violence was involved and played more than one role, which varied across different types of mental disorders.

We found physical violence, verbal abuse, or social humiliation played a role in predisposing a woman in developing mental disorder; emotional or psychological trauma played the most [Fig. 1]. We found emotional trauma resulting from negative remarks by their husband such as “you are black, you are not liked [by me], the girls outside are beautiful”, extramarital affair or sex with other woman, and second marriages were likely to lead to mental disorders. A woman who tried to harm herself deliberately several times because of her husband’s extramarital affairs alleged: “I have been hurt [emotionally] for the last year and a half. I have been tortured [emotionally], I have been dishonored”.

We found violence predisposed an individual for mental disorder even in later life. A psychiatrist narrated his experience of treating a sexual dysfunctional patient which he believed to be the result of her childhood sexual abuse. The patient took around 2 years to see a psychiatrist because no physician had ever explored her past traumatized life:

This girl was sexually abused at an early age. This girl is now married, got married. After marriage, her sexual life, she does not like it. [I] mean she becomes afraid when she sees a man; terror strikes her. When her husband comes to her or touches [her], she develops seizure like feature, right. She becomes tense, develop seizure like feature and try her best to avoid her husband.

We understood, as illustrated in Fig. 1, physical, verbal, and emotional violence worked as precipitators in many, while social in some cases of mental disorder. They played at least some role in precipitating acute stress disorder, depressive disorder, conversion disorders, schizophrenia, bipolar mood disorders, and obsessive compulsive disorder. A respondent with depression recalled her physical and verbal abuse and social suffering when she declined to marry a person 20 years older than her when she was 14 years old:

After breaking the marriage, meanwhile when I decline, then [I endured] beating with shoes, beating with broom, keeping without food, not allowed to sleep at home. My aunty came and said, “she is a prostitute, where [she] has become prostitute from”. Grand mother came and said, “pour some chili solution on her”, mother said, “kill her”, father came and beat me with stick, uncle came and say [the same], pulled my hair, [and] grandfather pushed me down.

We found psychological trauma resulted from refusal of marriage by a boyfriend as precipitator of mental disorders. A woman developed brief psychotic disorder when her boyfriend refused to marry her after a physical relationship. Premarital sex was not acceptable in this culture and was stigmatized. Consequent teasing by others worsened her suffering as her mother said:

People are not good; have said *habijabi* [demeaning words], “you have married off alone, gone out alone” and the men [who the girl was in relation and had sex] said, “I will be married off [by my parents]. I do not love you; do not have any relationship [with you]”. And father has forcibly brought her at night [because she went to her boyfriend house and living without marriage is not socially acceptable]. Various issues affected [her]. My daughter was healthy, that healthy daughter became crazy.

We found social violence or social humiliation stemmed from social politics and injustice as a precipitator in mental illness. A female school teacher was forced to ask for forgiveness from a lady who was in an extramarital affair with her husband. The unfair decision came from an arbitration meeting convened to resolve the issue where she could not establish her point, rather she was accused of defaming the lady engaged in the extramarital affair. This type of traditional dispute resolution is prevailing in Bangladesh which is often manipulated by local influential persons and delivers an unfair verdict [31]. Consequently, she developed acute stress disorder. She exclaimed: “The one who has broken my family she has not got any punishment. I have to seek forgiveness from her. [Interviewer: Is it more humiliating [then extramarital relationship]?] Yes.”

We found violence often worked as a maintenance factor; physical, verbal, emotional, and even social abuse or negligence played some role in maintaining conversion disorder, mental retardation with conduct disorders, bipolar mood disorders, obsession, and depression. Often parents, in laws, and very near ones hurled abusive language or physically hurt patients without understanding the impact. The ongoing abuse contributed to the maintenance of mental illness. A woman patient said how her recent episode recurred after being verbally abused by in laws:

In spite of talking in a sympathetic way, they said, “why you do not recover? You have destroyed my brother’s life. We have married our brother to such a women that my brother [has to suffer] throughout his whole life”.

A mismatched marital relationship where the wife had an outgoing personality often brought on physical violence by her husband and emotional suffering and contributed to the maintenance of mental disorders. A depressed lady English teacher who was often battered by her husband shared her feelings:

I do not have any thing in my life, sister [addressing the interviewer as her sister]. I have spent eight years of life like a prisoner, have not gone anywhere. Going out is a pastime, he does not have that. [Interviewer: Has your husband barred you to go out?]. [He] did not bar but who would I go with? He does not go anywhere, [he is] different.

We found mentally disordered people were considered as eccentric and down torn. People around them more often than not avoided them which were emotionally traumatic for the patient. This contributed to the deterioration of the existing mental disorder. A mother of a mentally retarded girl explained how her daughter felt when some visitors came to her house and did not talk to her:

That happens. When someone comes to my house and does not talk to her, then she feels bad in her heart. Again, when we go to somewhere to visit, when they talk to me but not to her, when a girl of her age comes but does not talk to her, she feels bad in her.

We found violence as a very common consequence of mental disorders; either by the patients towards themselves or by very close ones such as parents, brothers or sisters or even daughter or in laws and often by traditional healers. Patients with chronic mental disorders such as schizophrenia, bipolar mood disorders, mental retardation, and conduct disorders might face physical, verbal and social or sexual violence as a consequence of their condition [Fig. 1]. When the eccentric activities of a mentally disordered person went beyond tolerable limits; the caregivers might be burnt out and violence against the patient became a consequence irrespective of class and creed or understanding. Even the neighbors or people around often resorted to violence and parents used to endure that. A mother’s opinions on the beating of her bipolar mood disorder daughter by her neighbor was:

I do not blame them. [They] wanted to keep my daughter good taking into consideration my deplorable situation. [The neighbor might have thought] Alas! [The lady] did not get peace with her husband [husband is sick]; the daughter is now doing this. Let see if we can make her good by beating [because they did not consider her as ill rather with ill behaviors]

“All beat me” was the response of a girl suffering from schizophrenia while an educated daughter of a schizophrenic confessed how the close ones behaved with her mother:

My father has raised hand [to beat her], then when she was very crazy, making trouble, my younger sister has beaten her. Then my brother also raised hand on [her] body. When we kept her in our maternal uncles’ house, when she used to give trouble to them, maternal uncles have also beaten. We have our uncles where we are, they have beaten, beaten a lot.

When asked if the brothers and very close others understood that the patient was not mentally healthy, the daughter said: “[They] have understood, even then [they] have beaten [her]. Because, they understand that [she] is giving so much trouble, what would we do, let’s kill, give beating; if cured after being beaten, it’s a problem.”. The other reason of violence might be social stigma as the KI said:

I think they do this, two reasons behind this; one, they think if we can calm them this way before going to hospital we can save our prestige [face]. The second reason is that going to the doctor will make the issue public, that is why many guardians [come] at last after doing this [corporal punishment]. They try themselves.

We found that violence came with traditional treatment as a consequence. The treatment included beating with a broom and shoe, inflicting pain, holding burned chili or grass or flame or smoke in front of the nose and forcing inhalation to drive out the *bhoot* (spirit). A mother influenced by a relative consulted a *kabiraj* (a type of traditional healer) and described her experience of the treatment which caused a burn injury on the face of the patient:

A new cloth was soaked with mustard oil [and set on fire], then the arms [of the patient] were held [firmly so that she could not move away] and [the *kabiraj*] kept [the burning cloth in front of the nose and mouth of the patient] and said “if there is *alga* [possession] it will leave.” [Interviewer: [Had he] held the fire in front [of her face]?]. Yes. All these were burnt [pointing the burnt areas].

We found violence in the form of chaining used to restrain a mentally disordered person. Even at hospital it was in place. When asked why she fastened the chain around her daughter’s leg, a mother said:

[I] cannot keep her at home by any means. [We] got her admitted here [the NIMH hospital] for 8 days including today. One day [she] ran away [from here]. [Interviewer: where did she go?] Outside,[ she] will go away [outside of the hospital], this is the disease she is with, will go into hiding, [and] cannot be caught. [Interviewer: Did you chain her at home as well?] Yes, [I] keep her chained and a grill [an iron made structure used to secure the window or veranda] weighing 10 kg attached with it; she cannot go away with it.

We found psychological trauma as a consequence from avoidance, neglect, or negative attitude demonstrated to patients. Adults as well as children avoided or teased a person if s/he was known to have a mental disorder. It appeared that many times that violence, as mentioned above, did not fall under the category of gender violence because they were not related to gender identity but came as consequence of the mental disorder. It is never the less difficult to comment on this since we were only assessing the experience of women in the study.

Sometimes emotional trauma could come in a very different way as a consequence. One discussant said:

Violence can happen taking the opportunity of mental disorders such as some patients suffer from delusional disorders and under treatment, probably suspect husband. Now husbands take the opportunity. Because the wife already suspects him, everybody in society knows about it; the in laws know; all of the - girl’s father’s family know. The husband often indulges in extramarital relationships with two-three [women]. It is then seen that whatever the wife is saying, he is doing it, the wife also can sense it. The disease of the wife does not get better. He is doing the job but when told to all, nobody believes [it].

Apart from being victimized by others, some mental disorder patients often resort to violence against themselves, their family members or others because of the nature of the disease. When asked about a scar mark on a psychotic patient, her mother said:

Has bitten; bitten her, crazy [she] is. [Interviewer: has bitten herself?] Yes, has bitten herself. Has bitten all; her father, me, her brother, her sister and even her brother-in-law as well, did not spare from biting. Go this way, that way, if restrained, [she] bites.

Some patients developed mental disorders after their spouses with mental disorder indulged in violence against them. A gambler husband who has been beating his wife since their marriage, inflicted bruises over several areas and a head injury with bleeding inside as revealed by CT scan. He used to resort to violence when he needed money, when his wife protested about his gambling borrowing money from others or coming home late. Her sister described one incident: “After pushing her on *pacca* [concrete floor of the house] he kicked her. After that, [he] punched [her] head with both hands.”

Some violence did not appear to be related to mental disorders. Occasional slapping or smacking was usual in some family environments in Bangladesh and are not considered to be problematic. The following dialog was with a woman diagnosed as conversion disorder because of her mental agony related to financial issues:

Interviewer (I): How is the relationship with your husband?

Respondent (R): Relationship [with husband] is good overall; occasionally [he] slaps-smacks, that are problems [smiling].

I: Do you feel bad with these slaps or smacks? Is [it] painful for you?

R: No

I: Do you feel good with these slaps or smacks?

R: Yes, [I] feel good.

I: You feel good if slapped or smacked?

R: Yes

I: Why do you say feel well? Why do you feel good?

R: Feel well, that is it.

I: No, I mean why do you feel good if slapped or smacked?

R: Because *Mohabbat* [feeling of affection] increases.

A lady trainee psychiatrist supported this in the discussion:

They largely, to a great extent take it normally. There is less salt in curry, will be beaten [by husband], this is normal. They do not take it very seriously [that] I have become victim of violence and get upset, this is not the case. But, now, recently, there has been some change.

We came across a single type of violence which played multiple roles across different categories of mental disorders [Fig. 1] while more than one type were playing a single role in the same category of disorders. Some violence or conflict precipitated some category of mental disorder and then if continued contributed to its maintenance. Such type of disorders includes somatoform, dissociative or conversion disorders. A woman discussant said:

In conversion disorder, we see if there is some maintaining factors or continuous problem remaining, usually what happens in the case of tender aged young woman with conversion disorder, the main problem we get is some conflict with in laws, which [she] cannot overcome or cannot leave the in laws’ house. In the case, [in] case of conversion disorder, [violence] works as a maintaining factor.

A leading psychiatrist explains how the impact of violence varies across different disorders and their course.

For example, in depression, anxiety it can precipitate. This type of behavior can precipitate every disease. In the case of mental retardation, what happens, they become the big victims; many a times they are sexually abused,[and] physically abused. It is a sorrow that they cannot do anything and cannot protest or protect nothing happened [to the perpetrators]; we perhaps cannot do anything for them. They are the most neglected and suffered. In other instances can precipitate or perpetuate. Perhaps we can consider it as etiological factor, but gender violence is contributing as precipitating or perpetuating factors. [Interviewer: Can it be a co-incidence? Because it is quite common in our culture, can it be that there is no relationship but a co-incidence?]. That can be.

We found a patient who was diagnosed with bipolar disorder endured enormous physical abuse, psychological (husband’s spending money on having sex with other women] and verbal abuse by her husband and in laws. When asked by her mother, the patient narrated one of many incidence of her physical torture which in turn with psychological distress might result in her present mental disorder:

Mother, [he] watches TV all the night. I told him, can you please turn off the TV or switch off the light. I am going to sleep. He does not allow me to sleep. Then, because I have turned off the TV, he with rolling pin, used to prepare handmade bread hit me; the rolling pin was broken. Then, [my] head was swollen and the place [pointing a place in the head] became black, the present condition resulted from this; I cannot rememberanythingnow. I do not understand myself what I say to whom, do not understand.

## Discussion

We believe our qualitative study is the first one of its kind in understanding the experience of women suffering from mental disorders who came across some form of violence and exploring the relationship between violence and specific mental disorders in Bangladesh. The strength of the study is its findings extend beyond domestic or intimate partner violence and included a wide range of mental disorders. The context violence against women in Bangladesh is a male dominated society where violence against women often starts with marrying off when they are children. This is in line with the findings of others in similar cultural settings including Bangladesh [5, 32]. The negative influence of technology on the context of violence is also mentioned by others [33]. Corporal punishment and its cultural acceptability is common in other cultures as well [34]. Our findings of different types of violence with under reporting of sexual violence which often committed by close acquaintances were also reported elsewhere [35]. Our finding of mother in laws or sister in laws as instigators are consistent with other qualitative study in India and a mixed method study in Bangladesh [14, 36]. However, the findings of sons, brothers, sisters, or mothers as perpetrators were not mentioned previously in the literature as far as we know.

We found violence to be both predisposing and precipitating factors as well as a consequence or maintenance factors for mental disorders. The relationship is not straightforward as some quantitative or mixed method studies across different countries including Bangladesh have found [12, 19, 37]. In fact, the causes of mental disorders are multi factorial with complex interactions between factors [17]. Violence plays some role as other quantitative studies documented [9, 10, 38]. However, the effect varies across different disorders; sometimes it may predispose, many a time it may precipitate, it often results as a consequence and sometimes it is just a coincidence.

The strength of our study is that we looked in depth into how violence against women associated with specific mental disorders. Our study included all types of violence against women and all major categories of psychiatric disorders. The findings are supported by those of Naveed and Person on the triggers, risk factors and protective factors of violence in Bangladesh where violence would be a major modifiable risk factor [9, 39].

To reduce both the violence and mental disorders, we need to promote protecting factors and reduce facilitators through programmatic, healthcare resource strengthening, and regulatory measures. These views have the support of others [8, 9]. A qualitative exploration in a mixed method study found that social and spiritual support worked as a protective factor against mental disorders in women facing domestic violence [40, 41].

We found violence to be a common consequence of mental disorder, either committed by the patients on others or by others towards them. Other authors in quantitative and qualitative articles mentioned the issues [10, 42]. A qualitative study in India also found physical, social, and emotional violence facing patients with mental disorders [43]. However, all consequent violence women with mental disorder were facing was not because they were women as defined by others [8]; rather, they were victimized because of mental disorders as mentioned in other quantitative study [10]. They often faced violence because of “poor mental health literacy” among the general population and the caregivers [44]. The family and society usually take the role of sole care givers in Bangladesh, as mental services are grossly inadequate and poorly accessible [45]. Carers are often burnt out preventing harm caused by mentally disordered patients and may resort to violence as a mean of releasing their anger or of restraint and treatment because of supernatural beliefs [46, 47].

The main strength of our study in exploring the experience and understanding the role of violence against women with mental disorder was that all interviewers or analyst were qualified psychiatrists having a long familiarity with both the culture and the disease they were exploring. This allowed us to gather rich and valid information [24].

### Limitation of the study

We, the clinician researchers, conducted all the interviews and which may have hindered “frank discussion”. The long interviews taken place under a doctor patient relationship helped us to understand their experience without “fear, subconscious repression, and concealment” [28].

We did not interview other stakeholders such as general physicians or traditional healers. We did not visit the place where the incidence took place. We did not do it for various reasons; one was resource constraint and the other was our presence might raise undue attention which might be harmful to the respondent. However, we collected information from various sources and compared the emerging themes, finding our conclusions to be consistent across information sources. This we believe is the main essence of any qualitative research [48].

## Conclusions

We identified four key theoretical understandings. Many women in Bangladesh with mental disorders have been and continue to be exposed to violence. Gender violence played some role in many mental disorders but not in all. The roles of violence varied; sometimes it predisposed the disorder, while other times it precipitated or maintained the disorder. Sometimes it was just a coexistence or a consequence. The relationships were complex and sometimes depended on the type of mental disorders, nature, and intensity of the violence. Interestingly, not all violence faced by mentally disordered women was because they were women. Rather, they were facing violence because of the disease they were suffering from. The findings can be used in structuring culturally appropriate, but valid programs aimed at reducing both violence and the mental disorders they are associated with. Further mixed method research is recommended to enrich and quantify our understanding.

## Abbreviations

FGD: Focus group discussion
HARK: Humiliation, Afraid, Rape, and Kick
IDI: In-depth interview
KI: Key informant interview
NIMH: National Institute of Mental Health
WHO: World Health Organization
